# Patterns of Antibiotic Resistance in Community-Acquired Infections: A Study From a Tertiary Care Hospital

**DOI:** 10.7759/cureus.92904

**Published:** 2025-09-22

**Authors:** Aleeza Habib, Muhammad Rauf, Nandan Kumar Shah, Muhammad Umer Roohani, Muhammad Hamzatul Qadar Roohani, Arbaz Ahmad

**Affiliations:** 1 Department of Internal Medicine, Gujranwala Medical College, Gujranwala, PAK; 2 Department of General Medicine, King's College Hospital, London, GBR; 3 Department of General Medicine, B.P. Koirala Institute of Health Sciences (BPKIHS), Dharan, NPL; 4 Department of Emergency Medicine, Shaheed Uzair Mehmood Tehsil Headquarter (THQ) Hospital, Jand, PAK; 5 Department of Family Medicine, Maryam Nawaz Health Clinic, Kot Addu, PAK; 6 Department of Acute Medicine, Northampton General Hospital, Northampton, GBR

**Keywords:** antibiotic resistance, antimicrobial stewardship, community-acquired infections, escherichia coli, mrsa, multidrug-resistant organisms, pakistan

## Abstract

Background: Antibiotic resistance in community-acquired infections (CAIs) has emerged as a growing global concern with serious clinical and public health implications. Rising resistance to first-line therapies is reducing treatment effectiveness, increasing healthcare costs, and contributing to higher morbidity and mortality.

Objective: The objective is to evaluate the patterns of antibiotic resistance among bacterial isolates from CAIs in patients presenting to a tertiary care hospital and to assess their associated clinical outcomes to inform empirical therapy and support antimicrobial stewardship initiatives.

Methodology: A descriptive observational study was conducted at the Microbiology Department of Gujranwala Medical College, Gujranwala, Pakistan, from February 11, 2024, to February 11, 2025. The study included 560 individuals with suspected CAIs who tested positive for bacterial infection. Antimicrobial susceptibility testing was performed using the Kirby-Bauer disc diffusion method in accordance with Clinical and Laboratory Standards Institute guidelines. Data analysis was conducted using Statistical Package for the Social Sciences version 26 (IBM Corp., Armonk, NY).

Results: Of the 560 patients, 314 (56.07%) were men and 246 (43.93%) were women; 188 patients (33.57%) were aged 18-40 years. The most common specimen was urine (n = 234, 41.79%), followed by blood (n = 112, 20.00%). The predominant pathogens were *Escherichia coli* (n = 216, 38.57%) and *Staphylococcus aureus* (n = 104, 18.57%). Among *E. coli* isolates, 169 (78.24%) showed resistance to ciprofloxacin and 153 (70.83%) to ceftriaxone. Methicillin-resistant *S. aureus* (MRSA) was identified in 67 of 104 isolates (64.42%). Multidrug-resistant (MDR) organisms were detected in 188 patients (33.57%) and were significantly associated with treatment failure (38.30%), prolonged hospital stay (53.72%), antibiotic escalation (68.62%), and inhospital mortality (11.17%). Carbapenem-resistant infections were found in 32 patients (5.71%), with a mortality rate of 28.13% (n = 9).

Conclusion: This study demonstrates a high burden of MDR organisms in CAIs, with poor clinical outcomes, particularly in carbapenem-resistant and MRSA infections. These findings emphasize the urgent need for strengthened antimicrobial stewardship, continuous surveillance of resistance trends, and locally tailored empirical treatment guidelines in community healthcare settings.

## Introduction

Antibiotic resistance is one of the biggest risks to world health in the 21st century [1]. The World Health Organization estimates that antimicrobial resistance (AMR) is responsible for nearly 1.27 million deaths annually, with about 4.95 million deaths associated with resistant bacterial infections in 2019 alone [2]. If left unchecked, AMR could cause 10 million deaths per year by 2050 and result in a global economic loss of up to USD 100 trillion [3].

The emergence of resistance organisms has made it more challenging to control infections that were formerly readily treated with conventional antimicrobial medicines [4-6]. Although hospital-acquired infections get a lot of public attention, resistance trends in community-acquired diseases are just as concerning and need careful consideration [7]. Primary and emergency care settings often encounter these infections, which have an effect on patient outcomes and place a significant cost on healthcare systems, even if they originate outside of hospital settings [8]. Primary and emergency care settings often encounter these infections, which have a direct effect on patient outcomes and impose a significant cost on healthcare systems, estimated at USD 20 billion annually in direct healthcare costs in the United States alone [2,3,9].

Resistance has developed more quickly as a result of the extensive and often careless use of antibiotics in outpatient settings, self-medication, and a lack of regulatory oversight, especially in poor nations [10]. In South Asia, for example, studies report that the majority of antibiotics are purchased without a prescription, contributing to inappropriate use and incomplete treatment regimens [11]. Antibiotics are widely accessible without a prescription in many areas, which encourages abuse and unfinished treatment regimens, both of which aid in the selection and spread of resistant strains [12]. Additionally, empirical therapy without microbiological proof is still often used in clinical settings, which frequently results in the selection of incorrect antibiotics [13,14].

Due to inadequate sanitation, a lack of public health facilities, and a dense population, community-acquired infections (CAIs) are especially common throughout South Asia, including Pakistan. Nevertheless, there is still a dearth of local monitoring data on developments in antibiotic resistance [15,16]. As referral hubs for large catchment regions, tertiary care institutions are in a unique position to provide insights regarding patterns of resistance in the region [17]. Finding these trends is essential for revising treatment recommendations, directing empirical therapy, and influencing public health initiatives that promote antimicrobial stewardship [5,18].

Understanding the predominant resistance patterns among bacteria isolated from illnesses acquired in the community in a tertiary care hospital environment is the main goal of this investigation. The project intends to provide evidence-based clinical decision-making by highlighting the most prevalent infections and their drug susceptibility profiles via the analysis of regularly obtained microbiological data. The objective of this study was to evaluate the patterns of antibiotic resistance among bacterial isolates from CAIs in patients presenting to a tertiary care hospital.

## Materials and methods

Study design and setting

This descriptive observational study was conducted at the Medicine Department of Gujranwala Medical College, Gujranwala, Pakistan, over a one-year period from February 11, 2024, to February 11, 2025.

Selection criteria

Inclusion Criteria

Patients with a clinical suspicion of CAIs who presented to the emergency room and outpatient department, or were admitted to the hospital within 48 hours of arrival were included. Eligible participants were of all sexes and all ages. Only culture-positive samples were accepted, provided there was no history of hospitalization or antibiotic use within the preceding 72 hours.

Exclusion Criteria

Infections developing more than 48 hours after hospital admission were considered hospital-acquired and excluded. Patients with a history of hospitalization in the preceding 30 days, and those who had received antibiotics within the past 72 hours and duplicate isolates from the same illness episode were also excluded.

Sample size and sampling technique

Convenience sampling was used to recruit 560 consecutive patients from the outpatient, emergency, and early inpatient population suspected of having CAIs. This approach was chosen due to the single-center design and the objective of including all eligible patients who provided culture-positive samples over the one-year study period (from February 11, 2024, to February 11, 2025). The exploratory nature of the study and the aim to replicate real-world clinical and microbiological procedures precluded a formal a priori sample size calculation.

Based on hospital records, approximately 1,161 patients with suspected CAIs presented to the hospital during the study period, and our sample, therefore, represents ~48.2% of the total eligible population, providing substantial coverage for detecting prevalent resistance patterns. A post hoc sample size calculation confirmed that this cohort achieves >80% power to detect clinically meaningful differences in AMR among the major pathogens, minimizing the risk of type II error. The final sample size is also consistent with previous single-center observational studies examining AMR trends in comparable healthcare settings [19,20], supporting the validity and reliability of the findings.

Data collection

Clinical specimens were gathered in accordance with accepted microbiological practices and processed using standard aseptic methods. The Kirby-Bauer disc diffusion technique was used to identify the organisms and test for antibiotic susceptibility in accordance with the recommendations of the Clinical and Laboratory Standards Institute (2023 M100 Performance Standards for Antimicrobial Susceptibility Testing), under the supervision and guidance of the senior authors (MHQR and AA). Where appropriate, confirmatory phenotypic tests were carried out, particularly for suspected extended-spectrum beta-lactamase (ESBL)- or carbapenemase-producing organisms, as disc diffusion alone may not reliably detect all resistance mechanisms.

Multidrug resistance was defined as nonsusceptibility to at least one agent in three or more antimicrobial categories, in line with departmental consensus and senior oversight. Treatment failure was defined as persistence or worsening of clinical symptoms despite ≥72 hours of appropriate antimicrobial therapy. Antibiotic escalation was defined as the need to switch to a broader spectrum agent or combination therapy due to clinical deterioration or nonresponse, while deescalation was determined based on culture sensitivity, clinical improvement, and stewardship protocols. A hospital stay of greater than five days was categorized as prolonged hospitalization, and inhospital mortality was defined as death during the index admission.

Molecular typing and resistance gene identification, including detection of mecA, bla-TEM, and bla-NDM, were performed using polymerase chain reaction on a subset of multidrug-resistant (MDR) isolates, under the direct guidance of MHQR and AA, to strengthen resistance profiling.

Clinical data, including therapeutic outcomes, hospital stay, need for antibiotic modification, and mortality, were collected alongside microbiological findings. All information was recorded using a prestructured data collection form developed specifically for the study.

Statistical analysis

IBM Statistical Package for the Social Sciences version 26 (IBM Corp., Armonk, NY) was used for data entry and analysis. Descriptive statistics, including frequencies and percentages, were used to summarize the distribution of infections and antibiotic resistance patterns. Associations between antibiotic resistance and clinical outcomes (treatment failure, prolonged hospitalization, antibiotic escalation, and inhospital mortality) were assessed using chi-square tests or Fisher’s exact test where appropriate. A p value of <0.05 was considered statistically significant. This approach allowed the identification of patterns across infection types, demographic factors, and resistance profiles, supporting evidence-based clinical interpretation.

Ethical approval

The study received ethical clearance from the Institutional Review Board of Gujranwala Medical College, Gujranwala, under approval No. ME.IRB.684/GMC, dated 09/01/2024. Patient anonymity and confidentiality were strictly maintained, and all procedures adhered to the ethical principles of medical research involving human subjects.

## Results

A total of 560 patients with CAIs were enrolled in the study (Table 1). The majority of patients were between 18 and 40 years of age (n = 188, 33.57%), followed by those aged 41-60 years (n = 164, 29.29%), >60 years (n = 134, 23.93%), and <18 years (n = 74, 13.21%). Male patients constituted a slightly higher proportion of the cohort (n = 314, 56.07%) compared to female patients (n = 246, 43.93%). Most patients presented to the emergency department (n = 228, 40.71%), while 212 (37.86%) were managed in outpatient settings, and 120 (21.43%) were admitted within the first 48 hours of hospital stay. Regarding specimen types, urine samples were the most commonly submitted (n = 234, 41.79%), followed by blood (n = 112, 20.00%), wound swabs (n = 97, 17.32%), respiratory samples including sputum and throat swabs (n = 71, 12.68%), and other specimen types such as pus and ear discharge (n = 46, 8.21%).

**Table 1:** Demographic and clinical characteristics of patients (n = 560)

Variable	Category	Frequency (n)	Percentage (%)
Age group (years)	<18	74	13.21
	18-40	188	33.57
	41-60	164	29.29
	>60	134	23.93
Gender	Male	314	56.07
	Female	246	43.93
Clinical setting	Outpatient	212	37.86
	Emergency department	228	40.71
	Admitted <48 hours	120	21.43
Specimen type	Urine	234	41.79
	Blood	112	20.00
	Wound swab	97	17.32
	Respiratory (sputum/throat)	71	12.68
	Other (e.g., pus, ear discharge)	46	8.21

**Figure 1:**
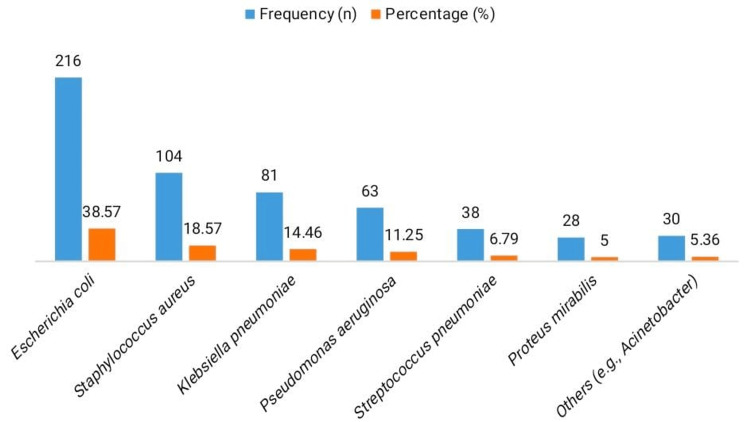
Distribution of bacterial isolates

Among the bacterial isolates obtained from culture-positive samples, *Escherichia coli* was the most prevalent organism, accounting for 216 cases (38.57%), as shown in Figure 1. *Staphylococcus aureus* was the second most frequent isolate (n = 104, 18.57%), followed by *Klebsiella pneumoniae* (n = 81, 14.46%) and *Pseudomonas aeruginosa* (n = 63, 11.25%). Less commonly isolated pathogens included *Streptococcus pneumoniae* (n = 38, 6.79%), *Proteus mirabilis* (n = 28, 5.00%), and other organisms such as Acinetobacter species (n = 30, 5.36%).

Resistance patterns varied significantly across bacterial species (Table 2). Among *E. coli* isolates (n = 216), high resistance was observed to ciprofloxacin (n = 169, 78.24%) and ceftriaxone (n = 153, 70.83%), while lower resistance was noted to amikacin (n = 32, 14.81%) and imipenem (n = 14, 6.48%). In *S. aureus* isolates (n = 104), 67 (64.42%) were oxacillin-resistant (i.e., methicillin-resistant *S. aureus*, MRSA), with resistance to clindamycin seen in 49 (47.12%) and vancomycin resistance detected in only three isolates (2.88%). *K. pneumoniae* (n = 81) showed resistance to cefotaxime in 53 isolates (65.43%), amikacin in 16 (19.75%), and imipenem in 8 (9.88%). *P. aeruginosa* (n = 63) demonstrated resistance to ceftazidime in 25 cases (39.68%), piperacillin-tazobactam in 19 (30.16%), and meropenem in 10 (15.87%).

**Table 2:** Antibiotic resistance patterns of key pathogens Each bacterial isolate was tested against multiple antibiotics. Therefore, resistance counts across different antibiotics are not mutually exclusive, and a single isolate may be resistant to more than one antibiotic. As a result, the total number of resistant cases for a pathogen may exceed the number of isolates MRSA: methicillin-resistant *Staphylococcus aureus*

Pathogen (n)	Antibiotic	Resistant (n)	Resistant (%)
*E. coli* (n = 216)	Ciprofloxacin	169	78.24
	Ceftriaxone	153	70.83
	Amikacin	32	14.81
	Imipenem	14	6.48
*S. aureus* (n = 104)	Oxacillin (MRSA marker)	67	64.42
	Clindamycin	49	47.12
	Vancomycin	3	2.88
*K. pneumoniae* (n = 81)	Cefotaxime	53	65.43
	Amikacin	16	19.75
	Imipenem	8	9.88
*P. aeruginosa* (n = 63)	Piperacillin-tazobactam	19	30.16
	Ceftazidime	25	39.68
	Meropenem	10	15.87

Infections caused by MDR organisms (n = 188, 33.57%) were significantly associated with worse clinical outcomes (Table 3). Among these, treatment failure occurred in 72 patients (38.30%), hospital stay >5 days in 101 (53.72%), need for antibiotic escalation in 129 (68.62%), and in-hospital mortality in 21 (11.17%). In contrast, patients infected with non-MDR organisms (n = 372, 66.43%) had notably better outcomes: treatment failure in 47 (12.63%), prolonged hospital stay in 86 (23.12%), antibiotic escalation in 68 (18.28%), and mortality in nine (2.42%). Similarly, ESBL-producing organisms (n = 142) were linked to high treatment failure (n = 58, 40.85%), longer hospitalizations (n = 85, 59.86%), antibiotic escalation (n = 102, 71.83%), and mortality (n = 17, 11.97%). The most adverse outcomes were observed in infections due to carbapenem-resistant organisms (n = 32), where 19 (59.38%) experienced treatment failure, 26 (81.25%) had prolonged hospital stays, 28 (87.50%) required antibiotic escalation, and nine (28.13%) died. MRSA infections (n = 67) also showed concerning outcomes with 24 (35.82%) treatment failures and six deaths (8.96%).

**Table 3:** Association between resistance profile and clinical outcomes (n = 560) Resistance categories and clinical outcomes are not mutually exclusive. A single isolate or patient may fall into multiple categories or outcomes; therefore, totals may exceed n = 560 MDR: multidrug-resistant; ESBL: extended-spectrum beta-lactamase; MRSA: methicillin-resistant *Staphylococcus aureus*

Resistance profile	Treatment failure, n (%)	Hospital stay >5 days, n (%)	Antibiotic escalation, n (%)	Inhospital mortality, n (%)
MDR pathogens (n = 188)	72 (38.30)	101 (53.72)	129 (68.62)	21 (11.17)
Non-MDR pathogens (n = 372)	47 (12.63)	86 (23.12)	68 (18.28)	9 (2.42)
ESBL producers (n = 142)	58 (40.85)	85 (59.86)	102 (71.83)	17 (11.97)
Carbapenem-resistant (n = 32)	19 (59.38)	26 (81.25)	28 (87.50)	9 (28.13)
MRSA (oxacillin-resistant) (n = 67)	24 (35.82)	36 (53.73)	42 (62.69)	6 (8.96)

A statistically significant association was observed between patient age and MDR infections (p = 0.003), with the highest MDR burden seen in the 18-60 age range (120 patients, 63.83%), shown in Table 4. Children under 18 years had significantly fewer MDR infections (n = 14, 7.45%). Although MDR infections were more frequent in men (n = 112, 59.57%) compared with women (n = 76, 40.43%), the difference was not statistically significant (p = 0.221). Clinical setting showed a strong association (p = 0.001), with emergency department patients (n = 87, 46.28%) and early admitted cases (n = 49, 26.06%) more likely to harbor MDR organisms compared to outpatients (n = 52, 27.66%). MDR pathogens were most commonly isolated from urine specimens (n = 84, 44.68%), followed by blood (n = 41, 21.81%) and wound swabs (n = 33, 17.55%), with specimen type significantly associated with resistance status (p = 0.038).

**Table 4:** Association between antimicrobial resistance and demographic/clinical variables (n = 560) p values are calculated using chi-square test ^*^Significant p values (p < 0.05) Effect size reported using Cramér’s V (interpretation: 0.1 = small, 0.3 = medium, 0.5 = large) MDR: multidrug-resistant

Variable	Category	MDR pathogens (n = 188)	Non-MDR pathogens (n = 372)	χ² (df)	p value	Effect size (Cramér’s V)
Age group	<18 years	14 (7.45)	60 (16.13)	13.87 (3)	0.003^*^	0.16 (small-medium)
	18-40 years	61 (32.45)	127 (34.14)			
	41-60 years	59 (31.38)	105 (28.23)			
	>60 years	54 (28.72)	80 (21.50)			
Gender	Male	112 (59.57)	202 (54.30)	1.50 (1)	0.221	0.05 (negligible)
	Female	76 (40.43)	170 (45.70)			
Clinical setting	Outpatient	52 (27.66)	160 (43.01)	13.46 (2)	0.001^*^	0.16 (small-medium)
	Emergency department	87 (46.28)	141 (37.90)			
	Admitted <48 hours	49 (26.06)	71 (19.09)			
Specimen type	Urine	84 (44.68)	150 (40.32)	10.27 (4)	0.038^*^	0.14 (small-medium)
	Blood	41 (21.81)	71 (19.09)			
	Wound swab	33 (17.55)	64 (17.20)			
	Respiratory	19 (10.11)	52 (13.98)			
	Other	11 (5.85)	35 (9.41)			

## Discussion

MDR microorganisms are very prevalent among CAIs, according to the study's results, with 33.57% of all culture-positive cases exhibiting MDR characteristics. The most often isolated organisms were *E. coli* (38.57%), *S. aureus* (18.57%), and *K. pneumoniae* (14.46%). These findings are consistent with previous research showing that *S. aureus* and *E. coli* are two of the most prevalent bacteria causing illnesses linked to healthcare, especially in young individuals and the elderly [21,22]. The alarming trend of fluoroquinolone resistance in community-acquired urinary tract infections reaching as high as 69% in some regions, especially in developing nations like Bangladesh, is reflected in the high rate of ciprofloxacin (78.24%) and ceftriaxone (70.83%) resistance among *E. coli* isolates in our study [23].

MRSA accounted for 64.42% of the isolates, with 2.88% displaying vancomycin resistance and 47.12% clindamycin resistance. A worrying increase in resistance trends is indicated by this higher MRSA burden, which exceeds previous community-based investigations where MRSA incidence often varied between 30% and 50% [24]. The rising prevalence of MRSA in outpatient settings points to a change in resistance from nosocomial to community settings, most likely as a result of inadequate infection control and uncontrolled antibiotic usage outside of hospitals.

Carbapenem-resistant organisms, mostly *K. pneumoniae* and *P. aeruginosa*, accounted for 5.71% (n = 32) of all isolates. Alarmingly bad outcomes were linked to these organisms: 28.13% died, 81.25% had extended hospital admissions, and 59.38% had treatment failure. This is in line with results from a systematic review and meta-analysis that showed that patients with carbapenem-resistant *K. pneumoniae* bacteremia had a considerably higher death rate, underscoring the crucial role that resistance plays in patient survival [25]. Notably, 25.36% (n = 142) of the patients in our analysis included ESBL-producing organisms, which were linked to a 40.85% treatment failure rate and an 11.97% fatality rate. This suggests that ESBL status significantly contributes to poor clinical outcomes in infections acquired in the community.

According to demographic analysis, MDR infections were more frequent among emergency department patients (46.28%, p = 0.001) and in adults aged 18-60 (63.83%, p = 0.003), which may indicate community transmission in mobile, active groups. Our findings show an increasing trend in resistance among working-age people, in contrast to earlier studies showing lower MDR incidence in younger groups [26]. Our research highlights the need for revised empirical recommendations and regional antimicrobial stewardship initiatives by demonstrating an increasing prevalence of resistance in CAIs, namely with MDR *E. coli*, MRSA, and carbapenem-resistant *K. pneumoniae*.

In comparison with national and international data, our findings reflect both similarities and concerning differences. The prevalence of MDR pathogens in our cohort (33.57%) is consistent with reports from South Asia, where MDR rates in CAIs often range between 30% and 40% [15]. However, the proportion of MRSA isolates (64.42%) in our study was notably higher than that reported in community settings of neighboring countries such as India and Bangladesh, where MRSA prevalence is typically 30%-50% [27]. Similarly, the rate of carbapenem resistance (5.71%) we observed, although lower than the 10%-12% reported in some tertiary hospitals in India and China, still highlights an emerging problem in Pakistan [28]. Alarmingly, fluoroquinolone resistance among *E. coli* isolates (78.24%) exceeded the WHO-reported regional average of 55%-65% in community-acquired urinary tract infections [29]. These comparisons indicate that while our local data follow broader regional resistance patterns, the higher burden of MRSA and fluoroquinolone-resistant *E. coli* in particular suggests an urgent need for targeted antimicrobial stewardship interventions in Pakistan.

It is important to note that vancomycin, while considered a last-line agent against resistant Gram-positive pathogens such as MRSA, carries risks of severe adverse drug reactions. Vancomycin-induced Drug Reaction with Eosinophilia and Systemic Symptoms syndrome, although rare, has been increasingly reported in recent years, with multiorgan involvement and significant mortality, particularly in elderly and immunocompromised patients [30]. These observations underscore the dual challenge of managing AMR while minimizing adverse drug effects, highlighting the need for safer alternatives and strict stewardship practices.

Strengths and limitations

This study's large and diverse sample, collected from emergency rooms and outpatient departments, is a major strength, allowing for a comprehensive analysis of MDR organisms in CAIs. The prospective design, strict inclusion criteria, and microbiological confirmation of isolates enhance the reliability and validity of the findings. Additionally, the study identifies key resistance patterns and associated clinical outcomes, providing valuable guidance for empirical antibiotic therapy.

However, there are some limitations. As a single-center study, the results may not be generalizable to regions with different pathogen profiles or prescribing practices. Although molecular characterization of carbapenemases (NDM, mecA, bla-TEM) was performed on a subset of MDR isolates, full molecular profiling of all resistant strains was not conducted, which limits the comprehensiveness of mechanistic insights. Furthermore, comorbidities and prior antibiotic use were not fully assessed, which could act as confounding factors influencing resistance trends. Future multicenter studies with broader molecular analyses are warranted to address these gaps.

## Conclusions

Our study shows that *S. aureus* and *E. coli* are the predominant MDR pathogens in CAIs, with particularly poor outcomes associated with carbapenem-resistant and ESBL-producing organisms. These findings have important clinical implications, as the high resistance to commonly prescribed antibiotics, such as ciprofloxacin, ceftriaxone, and clindamycin, challenges the effectiveness of standard empirical regimens and necessitates revisions to treatment protocols based on local resistance trends. From a public health perspective, the results underscore the urgent need for community- and region-specific antimicrobial stewardship programs and continuous surveillance to curb inappropriate antibiotic use. Limitations include the single-center design and lack of molecular characterization of resistance mechanisms, highlighting the need for multicenter studies with genomic analysis to better inform practice. In conclusion, this study provides critical evidence that resistance in CAIs is no longer a hospital-only problem but a growing community threat, demanding immediate action to optimize antibiotic policies and protect patient outcomes.
